# Association between metabolic syndrome and kidney cancer risk: a prospective cohort study

**DOI:** 10.1186/s12944-024-02138-5

**Published:** 2024-05-17

**Authors:** Lin Wang, Han Du, Chao Sheng, Hongji Dai, Kexin Chen

**Affiliations:** Department of Epidemiology and Biostatistics, Key Laboratory of Molecular Cancer Epidemiology, Key Laboratory of Prevention and Control of Human Major Diseases, Ministry of Education, National Clinical Research Center for Cancer, Tianjin Medical University Cancer Institute and Hospital, Tianjin Medical University, Tianjin, 300060 China

**Keywords:** Metabolic syndrome, Renal cancer, Hypertension, Central obesity, Dyslipidemia, Polygenic risk score

## Abstract

**Background:**

Kidney cancer has become known as a metabolic disease. However, there is limited evidence linking metabolic syndrome (MetS) with kidney cancer risk. This study aimed to investigate the association between MetS and its components and the risk of kidney cancer.

**Methods:**

UK Biobank data was used in this study. MetS was defined as having three or more metabolic abnormalities, while pre-MetS was defined as the presence of one or two metabolic abnormalities. Hazard ratios (HRs) and 95% confidence intervals (CIs) for kidney cancer risk by MetS category were calculated using multivariable Cox proportional hazards models. Subgroup analyses were conducted for age, sex, BMI, smoking status and drinking status. The joint effects of MetS and genetic factors on kidney cancer risk were also analyzed.

**Results:**

This study included 355,678 participants without cancer at recruitment. During a median follow-up of 11 years, 1203 participants developed kidney cancer. Compared to the metabolically healthy group, participants with pre-MetS (HR= 1.36, 95% CI: 1.06-1.74) or MetS (HR= 1. 70, 95% CI: 1.30-2.23) had a significantly greater risk of kidney cancer. This risk increased with the increasing number of MetS components (*P* for trend < 0.001). The combination of hypertension, dyslipidemia and central obesity contributed to the highest risk of kidney cancer (HR= 3.03, 95% CI: 1.91-4.80). Compared with participants with non-MetS and low genetic risk, those with MetS and high genetic risk had the highest risk of kidney cancer (HR= 1. 74, 95% CI: 1.41-2.14).

**Conclusions:**

Both pre-MetS and MetS status were positively associated with kidney cancer risk. The risk associated with kidney cancer varied by combinations of MetS components. These findings may offer novel perspectives on the aetiology of kidney cancer and assist in designing primary prevention strategies.

**Supplementary Information:**

The online version contains supplementary material available at 10.1186/s12944-024-02138-5.

## Background

Kidney cancer, a malignancy involving the urinary system, is the 14th most common cancer worldwide according to GLOBOCAN 2020 [1, 2]. Kidney cancer has posed a great burden on patients’ health and economic costs due to severe symptoms and difficulty in early detection and treatment [3]. Although tobacco, obesity, diabetes and hypertension are recognized as contributors in kidney cancer development [4], the cause of kidney cancer remains elusive. It is, therefore, important to identify additional indicators associated with kidney cancer for targeted preventive strategies in high-risk populations [5].

Metabolic syndrome (MetS) is a complex constellation of symptoms involving hypertension, central obesity, dyslipidemia, hypertriglyceridemia and hyperglycemia [6]. The increasing prevalence of MetS poses great challenges to public health [7]. Growing evidence suggests that MetS could be associated with overall cancer or site-specific cancers, such as liver cancer and colorectal cancer [8]. MetS is characterized by insulin resistance and chronic inflammatory state, possibly involved in cancer development and progression [9]. Emerging evidence links metabolic alterations and kidney cancer development from both animal experiments and population studies [10]. Under the condition of abnormal metabolism, tumor cells may gain more energy by increasing glycolysis and fatty acid oxidation, thereby promoting the development of kidney cancer [11]. To gather additional epidemiological evidence on the association linking MetS with kidney cancer, several population-based studies were carried out. However, most of the existing studies are retrospective, with limited sample sizes, and have shown inconsistent results [12–23]. Therefore, it is necessary to conduct further investigation into the relationship of MetS with kidney cancer risk.

Furthermore, environmental exposures and genetic factors may jointly affect the development of kidney cancer [24]. Until now, genome-wide association studies (GWAS) have uncovered genetic risk loci associated with kidney cancer [25]. The polygenic risk score (PRS), which is calculated by multiple genetic variants, serves as a comprehensive assessment of genetic susceptibility to diseases in individuals [26]. However, previous research has mainly assessed the single impact of PRS on kidney cancer [27]. The joint impact of MetS and PRS on kidney cancer remains unclear.

Based on a cohort of the European population, this study aimed to comprehensively examine the relationship of MetS and its components with kidney cancer risk and further explore the possible joint effect of MetS and PRS on kidney cancer. The findings of this study may offer novel perspectives on the etiology of kidney cancer and assist in designing primary prevention strategies.

## Methods

### Study design and participants

The study utilized data acquired from the UK Biobank, a prospective cohort, recruited over 500,000 individuals from 22 local assessment centers across the UK in 2006 - 2010 [28]. The cohort consisted of participants aged between 37 and 73 years. During the baseline assessment, individuals completed questionnaires, had physical measurements and had blood, urine and saliva samples gathered. This study excluded (a) participants with cancer at baseline (apart from non-melanoma skin cancer, ICD-10 (International Classification of Diseases, 10th revision) code (C44), (b) participants with missing MetS components, and (c) participants with missing covariates. The final analysis included 355,678 participants totally. The detailed flowchart of participant inclusion and exclusion was shown in Additional file: Figure S1.

### Assessment of MetS status

MetS was diagnosed based on the National Cholesterol Education Program Adult Treatment Panel III (NCEP-ATP III) criteria [7], with the presence of 3-5 of the subsequent metabolic abnormalities: (a) central obesity, defined as waist circumference [WC] ≥ 102 cm for males or WC ≥ 88 cm for females); (b) hypertension, a measured systolic blood pressure [SBP] ≥ 130 mmHg or diastolic blood pressure [DBP] ≥ 85 mmHg, and or taking antihypertensive drugs); (c) dyslipidemia, defined as high-density lipoprotein cholesterol [HDL-C] less than 1 mmol/L for males or less than 1.3 mmol/L for females, and or taking elevating HDL-C drugs; (d) hyperglycemia, defined as glycated haemoglobin [HbA1c] ≥ 42 mmol/mol or taking drugs for diabetes); (e) hypertriglyceridemia, defined as triglyceride [TG] ≥ 1.7 mmol/L or taking lowering TG drugs). Participants with one or two metabolic abnormalities were categorized into the pre metabolic syndrome (pre-MetS) group, while participants with none of the above-mentioned abnormalities were categorized into the metabolically healthy group.

WC was measured using a 200-cm tape measure (SECA). A HEM-907XL (Omron) was used to measure blood pressure twice after an interval of 5 minutes or more, and the mean of the two measured values was calculated [29]. The study chose a more stable marker, HbA1c, as a substitute to define hyperglycemia due to the low proportion (< 6%) of participants who had fasting glucose measurements. Medications for hypertension, low HDL-C, hypertriglyceridemia and hyperglycemia were defined as previously described [30].

### Ascertainment of outcomes

The study used an ICD-10 code of C64 to define kidney cancer. Cancer status was ascertained through hospital in-patient admission records, death or cancer registries data. This study followed the individuals from baseline enrollment until the earliest occurrence of the following events: first registration with kidney cancer, death, lost-of-follow-up, or the endpoint date (2021/01/31 in Scotland and 2020/02/29 in England and Wales).

### Covariates

This study adjusted for known kidney cancer risk factors and potential confounders, including sociodemographic information, lifestyle factors, dietary intake, and physical measurements. Covariate information was obtained through touch screen questionnaire and physical measurements. This study considered sociodemographic characteristics such as age, sex (male/female), race (white/others), education level (college or university degree/others) and the townsend deprivation index (TDI). Lifestyle factors consisted of smoking status (former or current/never) and drinking status (former or current/never). Diet intake was assessed using the food frequency questionnaire including vegetable intake (< 3 tablespoons a day/≥ 3 tablespoons a day), fruit intake (< 3 pieces a day/≥ 3 pieces a day), fish intake (< 2 times a week/≥ 2 times a week) and red meat intake (< 2 times a week/≥ 2 times a week). Physical measurement index included body mass index (BMI), which was calculated by weight (kg)/height (m)^2^.

### Polygenic risk score

The single nucleotide polymorphisms (SNPs) related to kidney cancer risk and their respective weights were obtained from previous GWAS studies [25, 31]. Standard PRS weights were used, and they corresponded to the log odds ratio (β) for each risk allele, with detailed information provided in Additional file: Table S1. A set of quality control procedures has been conducted on genomic data from the UK Biobank before the release of the processed data [32]. As GWAS studies on kidney cancer risk were conducted in European ancestry populations, self-reported white ancestry may not accurately represent European ancestry. Therefore, this study constructed the PRS based on the genotypes of individuals with Caucasian ancestry. Specifically, individuals were further excluded when they had missing genotypes, non-Caucasian ancestry, gender inconsistency, kinship relationships, or poor quality samples. A total of 107,797 participants were excluded, the subsequent analysis involved 247,881 remaining participants. In the PRS calculation, this study summed the weight of individual SNPs after each was multiplied by the allelic dosage, and then divided accumulated value by the number of SNPs.

$${PRS}_{j}=\sum_{i}\frac{{S}_{i}\times {G}_{ij}}{{M}_{j}}$$

where *M*_*j*_ is the number of SNPs observed for individual *j*, *S*_*i*_ is the weight of SNP *i* and G_*ij*_ is the allelic dosage of each SNP *i* in the genotype of individual *j*.

The PRS was classified into low (0-50th percentiles) and high (50-100th percentiles) genetic risk.

### Statistical analysis

Continuous variables were presented as mean (standard deviation), while categorical variables were shown as number (percentage). The study performed one-way analysis of variance (ANOVA) or Chi-squared test to compare baseline information among the metabolically healthy, pre-MetS and MetS groups.

The study constructed Cox regression models to evaluate the relationship between MetS status, MetS components and kidney cancer risk. Model 1 included age and sex, while Model 2 further included race, education level, TDI and dietary intake; Model 3, the full model, additionally incorporated BMI based on Model 2. The associations of exposures were presented by hazard ratios (HRs) and 95% confidence intervals (95% CIs). The study further investigated the associations between the combinations of main MetS component and kidney cancer risk. The Schoenfeld test showed no violation of the proportional hazards assumption. Prespecified subgroup analyses were conducted according to sex (male/female), age (≥ 60 years old/< 60 years old), BMI (≥ 30 kg/m^2^/25 – 29.9 kg/m^2^/< 25 kg/m^2^), smoking status (current/former/never), and drinking status (current/ former/never). The study used the Wald test to assess the significance of an interaction term. To evaluate the nonlinear relationship of each MetS component with kidney cancer risk, restricted cubic splines (RCS) with four knots (5%, 35%, 65%, and 95%) was applied. Additionally, in the Caucasian population, this study used multivariable Cox regression to evaluate the relationship of MetS, PRS with kidney cancer risk.

Sensitivity analyses were conducted to confirm the results stability. First, to eliminate the effect of reverse causality, individuals who was follow-up less than 2 years were excluded; Second, the study excluded individuals who had outliers for MetS components. Values below 1% or above 99% quantile were regarded as outliers. Third, the study excluded individuals with diabetes at recruitment. Fourth, individuals with kidney cancer ascertained through the death registries data were excluded.

A 2-sided *P* value < 0.05 was determined as statistically significant and all statistical analyses were used with R software (4.2.1).

## Results

### Baseline characteristics

In total, 355,678 participants were involved. The mean (SD) age was 56.3 (8.1) years and the proportion of males was 46.8%. Participants were stratified into three categories based on the MetS status: the metabolically healthy group (16.1%), the pre-MetS group (56.3%) and the MetS group (27.6%) (Table 1). Compared with the metabolically healthy group, participants with pre-MetS or MetS tended to be older, male and former or never drinkers, with a lower level of education and a history of former or current smoking. Individuals with MetS tended to have a greater proportion of non-white ancestry, a BMI≥ 30 kg/m^2^ and a greater TDI than those from the pre-MetS and metabolically healthy groups. As expected, the pre-MetS and MetS groups had elevated WC, DBP, SBP, TG and HbA1c levels, along with reduced HDL-C levels. Additionally, the proportion of taking statins, antidiabetic medications and antihypertensive medications was 35.8%, 7.1% and 40.8% respectively in the MetS group. For each MetS component, hypertension showed the highest prevalence rate (69%), with hyperglycemia having the lowest prevalence rate (8%) (Additional file: Figure S2). Furthermore, this study compared the baseline information between participants with all MetS components and those with missing components, and found there were no difference between the two groups (Additional file: Table S2).

**Table 1:** Baseline characteristics of the study population

Characteristic	Total	Metabolically healthy	pre-MetS	MetS	*P*
*N* (%)	355678 (100)	57303 (16.1)	200182 (56.3)	98193 (27.6)	
Age, mean (SD), years	56.3 (8.1)	52.0 (7.6)	56.5 (8.0)	58.2 (7.6)	<0.001
Gender, *N* (%)					<0.001
Female	189263 (53.2)	40557 (70.8)	101219 (50.6)	47487 (48.4)	
Male	166415 (46.8)	16746 (29.2)	98963 (49.4)	50706 (51.6)	
Ethnicity, *N* (%)					<0.001
Nonwhite	16263 (4.6)	2518 (4.4)	8372 (4.2)	5373 (5.5)	
White	339415 (95.4)	54785 (95.6)	191810 (95.8)	92820 (94.5)	
Townsend deprivation index, mean (SD)	-1.4 (3.0)	-1.5 (3.0)	-1.6 (3.0)	-1.1 (3.2)	<0.001
Education level, *N* (%)					<0.001
College or university degree	119056 (33.5)	25601 (44.7)	68638 (34.3)	24817 (25.3)	
Others	236622 (66.5)	31702 (55.3)	131544 (65.7)	73376 (74.7)	
Smoking status, *N* (%)					<0.001
Never	195961 (55.1)	35377 (61.7)	112358 (56.1)	48226 (49.1)	
Former	122917 (34.6)	16591 (29.0)	67694 (33.8)	38632 (39.3)	
Current	36800 (10.3)	5335 (9.3)	20130 (10.1)	11335 (11.5)	
Drinking status, *N* (%)					<0.001
Never	14330 (4.0)	1800 (3.1)	6915 (3.5)	5615 (5.7)	
Former	12060 (3.4)	1622 (2.8)	5752 (2.9)	4686 (4.8)	
Current	329288 (92.6)	53881 (94.0)	187515 (93.7)	87892 (89.5)	
BMI category, *N* (%)					<0.001
<25kgm^2^	118405 (33.3)	38978 (68.0)	73423 (36.7)	6004 (6.1)	
25-29.9 kg/m^2^	152018 (42.7)	17365 (30.3)	98116 (49.0)	36537 (37.2)	
≥30 kg/m^2^	85255 (24.0)	960 (1.7)	28643 (14.3)	55652 (56.7)	
BMI, mean (SD)	27.4 (4.7)	23.8 (2.7)	26.5 (3.8)	31.3 (4.8)	<0.001
WC, mean (SD), cm	90.2 (13.4)	78.6 (8.7)	87.9 (11.0)	101.7 (11.9)	<0.001
SBP, mean (SD), mm Hg	137.7 (18.5)	118.1 (8.1)	140.3 (17.8)	144.0 (16.8)	<0.001
DBP, mean (SD), mm Hg	82.3 (10.1)	73.0 (6.3)	83.3 (9.7)	85.5 (9.8)	<0.001
HDL-C, mean (SD), mmol/l	1.5 (0.4)	1.7 (0.3)	1.5 (0.4)	1.2 (0.3)	<0.001
Triglyceride, mean (SD), mmol/l	1.7 (1.0)	1.0 (0.3)	1.6 (0.8)	2.5 (1.2)	<0.001
HbA1C, mean (SD), mmol/mol	36.0 (6.6)	33.7 (3.2)	34.8 (4.2)	39.7 (9.8)	<0.001
Statins (Yes, %)	56448 (15.9)	0 (0.0)	21251 (10.6)	35197 (35.8)	<0.001
Diabetes medications (Yes, %)	7662 (2.2)	0 (0.0)	714 (0.4)	6948 (7.1)	<0.001
Hypertension medications (Yes, %)	71698 (20.2)	0 (0.0)	31660 (15.8)	40038 (40.8)	<0.001

*P* values were calculated by Chi-squared test for categorical data and one-way analysis of variance for parametric data

*Abbreviations*: *pre-MetS* pre metabolic syndrome, *MetS* metabolic syndrome, *BMI* body mass index, *WC* waist circumference, *SBP* systolic blood pressure, *DBP* diastolic blood pressure, *HDL-C* high-density lipoprotein cholesterol, *SD* standard deviation, *N* Number

### MetS and kidney cancer

**Fig. 1:**
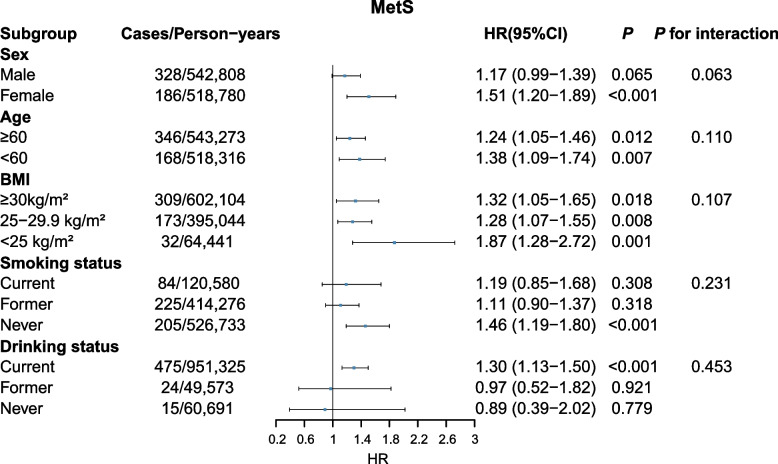
Association of MetS with risk of kidney cancer stratified by different subgroups

Compared with non-MetS (metabolically healthy + pre-MetS) group, MetS status showed a positive association with kidney cancer risk (HR= 1.28, 95% CI: 1.11-1.46) (Table 2). Additionally, there was a higher HR for MetS and pre-MetS (pre-MetS: HR= 1.36, 95% CI: 1.06-1.74; MetS: HR= 1.70, 95% CI: 1.30-2.23) compared to metabolically healthy group to develop kidney cancer. Compared to individuals with no MetS components, those with one to five components had HRs of 1.26 (0.97-1.64), 1.52 (1.17-1.98), 1.64 (1.24-2.16), 2.02 (1.49-2.75), and 2.65 (1.82-3.86), successively (*P* for trend < 0.001). The results remained consistent across all sensitivity analyses (Additional file: Table S3-S6). Subgroup analyses results demonstrated that the associations of MetS with kidney cancer risk according to sex, age, BMI and smoking status were consistent. MetS exhibited a greater association with kidney cancer risk among current drinkers than former/never drinkers. However, no evidence of interaction was observed within these five groups (all *P* for interaction > 0.05, Fig. 1).

**Table 2:** Associations between MetS and kidney cancer risk

MetS or component	Cases/Person-years	Incidence rates(per 100,000)	HR (95% CI)							
			Crude	*P*	Model1	*P*	Model2	*P*	Model3	*P*
MetS										
No	689/2,824,781	24.39	Reference		Reference		Reference		Reference	
Yes	514/1,061,588	48.42	1.99 (1.77-2.23)	<0.001	1.63 (1.46-1.83)	<0.001	1.57 (1.40-1.76)	<0.001	1.28 (1.11-1.46)	<0.001
MetS stage										
Metabolically healthy	74/632,519	11.7	Reference		Reference		Reference		Reference	
pre-MetS	615/2,192,263	28.05	2.40 (1.89-3.06)	<0.001	1.55 (1.21-1.98)	<0.001	1.51 (1.18-1.93)	0.001	1.36 (1.06-1.74)	0.016
MetS	514/1,061,588	48.42	4.15 (3.25-5.30)	<0.001	2.41 (1.88-3.09)	<0.001	2.26 (1.76-2.90)	<0.001	1.70 (1.30-2.23)	<0.001
Hypertension										
No	196/1,209,479	16.21	Reference		Reference		Reference		Reference	
Yes	1007/2,676,890	37.62	2.32 (1.99-2.71)	<0.001	1.49 (1.27-1.74)	<0.001	1.45 (1.24-1.70)	<0.001	1.29 (1.10-1.51)	0.002
Central obesity										
No	654/2,618,222	24.98	Reference		Reference		Reference		Reference	
Yes	549/1,268,148	43.29	1.74 (1.55-1.94)	<0.001	1.66 (1.48-1.86)	<0.001	1.58 (1.41-1.77)	<0.001	1.22 (1.04-1.42)	0.013
Dyslipidemia										
No	840/3,107,014	27.04	Reference		Reference		Reference		Reference	
Yes	363/779,356	46.58	1.72 (1.52-1.95)	<0.001	1.87 (1.65-2.11)	<0.001	1.83 (1.62-2.08)	<0.001	1.63 (1.43-1.86)	<0.001
Hypertriglyceridemia										
No	470/2,033,870	23.11	Reference		Reference		Reference		Reference	
Yes	733/1,852,500	39.57	1.72 (1.53-1.93)	<0.001	1.24 (1.10-1.40)	<0.001	1.19 (1.06-1.35)	0.004	1.05 (0.93-1.18)	0.472
Hyperglycemia										
No	1045/3,584,630	29.15	Reference		Reference		Reference		Reference	
Yes	158/301,740	52.36	1.81 (1.53-2.14)	<0.001	1.35 (1.14-1.60)	<0.001	1.32 (1.11-1.56)	0.002	1.09 (0.92-1.31)	0.315
No.MetS components										
0	74/632,518	11.7	Reference		Reference		Reference		Reference	
1	256/1,153,295	22.2	1.90 (1.47-2.46)	<0.001	1.34 (1.03-1.74)	0.028	1.32 (1.02-1.71)	0.038	1.26 (0.97-1.64)	0.085
2	359/1,038,968	34.55	2.96 (2.30-3.80)	<0.001	1.77 (1.37-2.29)	<0.001	1.71 (1.32-2.20)	<0.001	1.52 (1.17-1.98)	0.002
3	283/677,179	41.79	3.58 (2.77-4.63)	<0.001	2.10 (1.62-2.72)	<0.001	1.98 (1.53-2.58)	<0.001	1.64 (1.24-2.16)	<0.001
4	168/306,181	54.87	4.71 (3.58-6.19)	<0.001	2.78 (2.10-3.67)	<0.001	2.61 (1.97-3.45)	<0.001	2.02 (1.49-2.75)	<0.001
5	63/78,229	80.53	6.93 (4.95-9.70)	<0.001	3.87 (2.75-5.44)	<0.001	3.62 (2.57-5.11)	<0.001	2.65 (1.82-3.86)	<0.001
P for trend			<0.001		<0.001		<0.001		<0.001	

Model1: Adjusted for age and sex

Model2: Adjusted for age, sex, ethnicity, townsend deprivation index, education level, smoking status, alcohol status and intakes of vegetables, fruit, fish, and red meat

Model3: Adjusted as model 2 plus body mass index

*Abbreviations*: *MetS* metabolic syndrome, *HR* hazard ratio, *95% CI* 95% confidence interval, *No.* Number, *Crude* Univariable cox regression

### MetS components and kidney cancer

All five MetS components (binary classification) were associated with kidney cancer risk across Model 1 and Model 2 (all *P* < 0.05). However, after additionally adjusting for BMI, only hypertension (HR= 1. 29, 95% CI: 1.10-1.51), central obesity (HR= 1. 22, 95% CI: 1.04-1.42) and dyslipidemia (HR= 1. 63, 95% CI: 1.43-1.86) remained significantly associated with kidney cancer (Table 2). This study further explored the relationships of main MetS component combinations with kidney cancer risk (Table 3). In Model 3, the highest HRs for kidney cancer risk based on component combinations were BP + HDL: 2.34 (1.44-3.81) for pre-MetS and BP + HDL + WC: 3.03 (1.91-4.80) for MetS, respectively. The results of sensitivity analyses were consistent (Additional file: Table S7 and S8). In the subgroup analyses (Additional file: Figure S3-S7), hypertriglyceridemia was predominantly associated with a greater risk of kidney cancer in women (HR= 1.37, 95%CI: 1.12-1.68) than in men (HR= 0.90, 95%CI: 0.77-1.05, *P* for interaction = 0.002, Additional file: Figure S3). The associations of central obesity, hyperglycemia, dyslipidemia and hypertension with kidney cancer risk were not substantially different based on these subgroups (all *P* for interaction > 0.05, Additional file: Figure S4-7).

**Table 3:** Adjusted HRs for kidney cancer with MetS component combinations

No. of MetS components	Cases/Person-years	Incidence rates (per 100,000)	Combinations of MetS components	Adjusted HR (95% CI)	*P*
Metabolically healthy	74/632,518	11.70	None	Reference	
(0 component)					
pre-MetS(1-2)	193/806,188	23.94	BP	1.26 (0.96-1.65)	0.095
(1-2 components)	13/71,263	18.24	HDL	2.01 (1.11-3.62)	0.021
	30/183,074	16.39	TG	0.91 (0.59-1.39)	0.652
	19/85,637	22.19	WC	1.53 (0.92-2.56)	0.105
	21/56,582	37.11	BP + HDL	2.34 (1.44-3.81)	0.001
	200/558,960	35.78	BP + TG	1.38 (1.05-1.82)	0.022
	96/244,613	39.25	BP + WC	1.75 (1.26-2.43)	0.001
	14/59,167	23.66	HDL + TG	1.54 (0.87-2.74)	0.138
	19/62,870	30.22	TG + WC	1.52 (0.91-2.54)	0.114
MetS(3-5)	78/141,210	55.24	BP + HDL + TG	2.33 (1.68-3.23)	<0.001
(3-5 components)	28/54,037	51.82	BP + HDL + WC	3.03 (1.91-4.80)	<0.001
	137/355,311	38.56	BP + WC + TG	1.35 (0.98-1.84)	0.065
	10/15,704	63.68	BP + WC + HbA1c	2.35 (1.19-4.64)	0.014
	14/48,616	28.80	HDL + WC + TG	1.74 (0.97-3.12)	0.064
	14/47,317	29.59	BP + TG + HbA1c	0.96 (0.54-1.72)	0.897
	106/196,662	53.90	BP + HDL + WC +TG	2.19 (1.57-3.05)	<0.001
	41/73,598	55.71	BP + WC + TG + HbA1c	1.61 (1.06-2.45)	0.025
	63/78,229	80.53	BP + HDL + WC + TG + HbA1c	2.65 (1.81-3.89)	<0.001

HR was adjusted for age, sex, ethnicity, townsend deprivation index, education level, smoking status, alcohol status, intakes of vegetables, fruit, fish, and red meat and body mass index

Note: only combinations with >10 events were presented

*Abbreviations*: *pre-MetS* pre metabolic syndrome, *MetS* metabolic syndrome, *BP* blood pressure, *HDL* high-density lipoprotein cholesterol, *TG* triglycerides, *WC* waist circumference, *No.* Number, *HR*, hazard ratio, *95% CI* 95% confidence interval

**Fig. 2:**
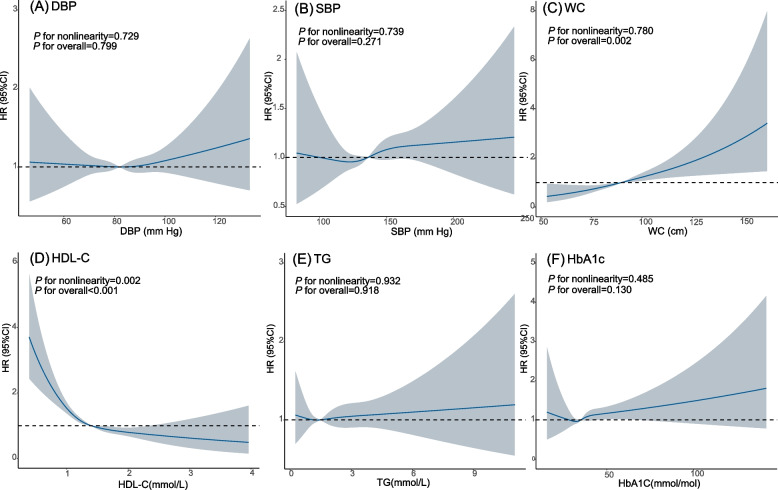
Restricted cubic spline analysis for the associations between MetS components and kidney cancer risk

When examining the non-linear relationship of MetS components (continuous) with kidney cancer risk, only HDL-C and WC demonstrated significant associations with kidney cancer risk (HDL-C: *P* for overall < 0.001; WC: *P* for overall = 0.002). The study revealed an L-shaped relationship between HDL-C and kidney cancer risk (*P* for nonlinearity = 0.002). Higher WC exhibited a positive association with kidney cancer risk, without evidence of nonlinearity (*P* for nonlinearity = 0.780). Modelling the MetS component with the RCS suggested no association between DBP, SBP, TG or HbA1c and kidney cancer risk (all *P* for overall > 0.05, all *P* for nonlinearity > 0.05, Fig. 2).

### MetS, PRS and kidney cancer

The study further explored the relationship of PRS with kidney cancer risk, along with evaluating the combined effect of MetS and PRS across multivariable Cox models. The results demonstrated that individuals at high genetic risk had a greater kidney cancer risk (HR = 1.36, 95% CI: 1.19-1.56) when in comparison to those at low genetic risk. In Model 3, each 1-SD increase in PRS level corresponded to a 16% rise in kidney cancer risk (HR = 1.16, 95% CI: 1.09-1.24). Compared with participants with non-MetS and low PRS, those with MetS and high PRS had a significantly greater kidney cancer risk (HR= 1.74, 95% CI: 1.41-2.14) (Table 4).

**Table 4:** Risk of kidney cancer according to the joint effect of MetS and PRS

	Cases/Person-years	Incidence rates (per 100,000)	HR (95% CI)					
			Model1	*P*	Model2	*P*	Model3	*P*
PRS								
low PRS	369/1,356,865	27.20	Reference		Reference		Reference	
high PRS	502/1,355,276	37.04	1.36 (1.19-1.56)	<0.001	1.36 (1.19-1.56)	<0.001	1.36 (1.19-1.56)	<0.001
continuous			1.16 (1.09-1.24)	<0.001	1.16 (1.09-1.24)	<0.001	1.16 (1.09-1.24)	<0.001
Joint effect of MetS and PRS								
No MetS/low PRS	213/989,257	21.53	Reference		Reference		Reference	
MetS/low PRS	156/367,608	42.44	1.65 (1.34-2.03)	<0.001	1.57 (1.27-1.93)	<0.001	1.28 (1.02-1.60)	0.032
No MetS/high PRS	289/986,870	29.28	1.36 (1.14-1.62)	<0.001	1.36 (1.14-1.62)	<0.001	1.36 (1.14-1.62)	<0.001
MetS/high PRS	213/368,405	57.82	2.24 (1.85-2.71)	<0.001	2.14 (1.77-2.59)	<0.001	1.74 (1.41-2.14)	<0.001

Model1: Adjusted for age and sex

Model2: Adjusted for age, sex, townsend deprivation index, education level, smoking status, alcohol status and intakes of vegetables, fruit, fish, and red meat

Model3: Adjusted as model 2 plus body mass index

low PRS referred to 0-50th percentiles; high PRS referred to 50-100th percentiles

*Abbreviations*: *MetS* metabolic syndrome, *PRS* polygenic risk score, *HR* hazard ratio, *95% CI* 95% confidence interval

## Discussion

The study found a positive relationship between both pre-MetS and MetS and kidney cancer risk, with the risk increased corresponding to the MetS components number. The risk associated with kidney cancer varied by MetS components combinations. Additionally, there was an obviously higher kidney cancer risk in individuals with high PRS and MetS, suggesting that PRS and MetS could exert a joint effect on kidney cancer risk.

Limited prospective studies have examined the relationship between MetS and kidney cancer risk, showing inconsistent conclusions. Kailuan [16] and Me-Can [17] cohort studies results demonstrated a positive association between MetS and kidney cancer risk, which consisted with results in this study. Another SMART cohort study conducted among individuals with cardiovascular diseases (CVD) observed no relationship between MetS and kidney cancer, possibly due to metabolic alterations caused by CVD [18]. Several retrospective cohort studies [12, 14, 19, 20], case–control studies [13, 21, 22], cross-sectional study [23], and meta-analysis [15] found that MetS could increase kidney cancer risk. Nevertheless, most studies have retrospective designs, limited sample sizes, and low statistical efficiency. Several studies suggested positive associations between pre-MetS and heart disease [33, 34], however, few research studied on the relationship of pre-MetS with kidney cancer risk. The study revealed an increase in kidney cancer risk among individuals suffering from pre-MetS, indicating the necessity of implementing intervention measures to prevent kidney cancer among this population.

Among the five MetS components, the study found that hypertension, central obesity and dyslipidemia were linked to kidney cancer at a higher risk. Hypertension is a common contributing factor to kidney cancer [35]. Unlike general obesity, central obesity exhibits excessive accumulation of abdominal fat. One cohort study exhibited a 1.32-fold increase in kidney cancer risk in participants suffering from central obesity, with the risk increasing by increasing WC [36], which consisted with findings in the study. For dyslipidemia, previous reports showed a positive association for low HDL-C with kidney cancer risk [37], similar to the findings of present study. Furthermore, this study found an L-shaped nonlinear relationship between HDL-C level and kidney cancer risk. Similar nonlinear relationships between HDL-C level and all-cause mortality were identified [38]. There were no statistically significant associations between hypertriglyceridemia or hyperglycemia and kidney cancer risk in this study, consistent with findings from a case-control study using the Kailuan database [13]. However, some studies have reported that high TG levels increased kidney cancer risk [12, 39]. The heterogeneity in the results could be attributed to different studied populations and adjusted factors. A case-control study from Taiwan also reported no associations between hyperglycemia and kidney cancer [40]. In this study, various effects of MetS component combinations on kidney cancer risk were identified. Notably, the risks of participants in some pre-MetS groups (e.g., high BP + low HDL, HR=2.34) were higher than those in some MetS groups (e.g., BP + WC + TG, HR=1.35). This finding suggests that individuals with high BP and low HDL should be targeted for early prevention and management, even if they do not satisfy the criteria for diagnosing MetS. Kidney cancer risk was highest among individuals with a combination of high BP, low HDL and increased WC in the MetS group. The observed association is plausible because only these three factors showed significant associations with kidney cancer risk in former MetS component analyses. Moreover, hypertension, dyslipidemia or central obesity might interact through common pathophysiological pathways (such as insulin resistance) [41] in tumorigenesis. Further investigations are required to clarify the potential mechanisms linking MetS component combinations to kidney cancer development. The genetic variants identified through GWAS can be utilized for constructing PRS to identify high-risk populations for preventing diseases [42]. In this study, kidney cancer risk substantially increased in participants with MetS and high PRS. Considering the relative immutability of genetic risk, intervention measures aimed at populations with MetS and high PRS could be efficient to lower kidney cancer incidence.

Although the pathogenesis of MetS and kidney cancer remains unclear, there are some potential mechanisms for MetS to increase kidney cancer risk. Insulin resistance promotes reactive oxygen species production, leading to DNA damage and facilitating malignant transformation [43]. Hyperinsulinemia elevates type 1 insulin-like growth factor (IGF-1), activating downstream signaling pathways such as PI3K/Akt/mTOR, which promotes cell proliferation, inhibits apoptosis, and induces carcinogenesis [44]. Obesity changes the levels adipocyte-secreted hormone, among which adiponectin inhibits cell proliferation, leptin stimulates cell proliferation and facilitates invasion and migration [45]. Additionally, obesity can lead to an increase of pro-inflammatory factors, which may inhibit the immune system function and promote tumor growth [46]. Furthermore, hypertension may affect the development of kidney cancer through chronic renal hypoxia, lipid peroxidation and angiotensin system disorders [47–49]. In the presence of multiple coexisting components, various mechanisms may act synergistically to influence kidney cancer risk. Hypertension and dyslipidemia, mechanistically linked, could exacerbate atherosclerosis and impact vascular endothelial growth factor, thereby influencing tumor growth [41]. Hyperglycemia and hypertension share common mechanisms, such as oxidative stress, which may interact with other pathways to accelerate the development of kidney cancer [50]. However, biological mechanisms underlying the effects of MetS component combinations on kidney cancer are not clear, and further research is needed.

### Study strengths and limitations

A significant strength of this study is its comprehensive and detailed measurements of metabolic factors within a large prospective cohort design. The study followed up with > 1000 cases of kidney cancer, which provided high statistical power and allowed for detailed examinations of subgroups. Additionally, the risk associated with kidney cancer varied by combinations of MetS component in this study. High-risk populations were characterized by the coexistence of multiple components, such as the combination of ‘BP+HDL+ WC’. Furthermore, this study found the combined effect of PRS and MetS on kidney cancer initially.

Several limitations existed in this study. First, MetS components were measured only once at baseline. Therefore, the dynamic trends of metabolic risks cannot be evaluated; Second, the impact of confounding factors cannot be completely eliminated, though the models were adjusted for a variety of factors; Third, kidney cancer cases (*N*=19) identified by death registries data may overestimate follow-up period, potentially influencing the findings. Finally, the extrapolation of the results is limited since study population were mostly of European ancestry.

## Conclusion

In this study, both pre-MetS and MetS were associated with higher risk of kidney cancer. Combinations of MetS components had various effects on kidney cancer. BP, HDL and WC were among the strongest metabolic risk factors for kidney cancer. Consequently, inclusion of the population’s metabolic status becomes imperative in designing primary prevention strategies for kidney cancer. Additionally, the combination of MetS and PRS may better predict the kidney cancer risk in population, facilitating early prevention efforts.

### Supplementary Information

Supplementary Material 1.

## Data Availability

UK Biobank data is available at https://www.ukbiobank.ac.uk/. This research was conducted under approved project #76092.

## Abbreviations

MetS: Metabolic syndrome
pre-MetS: Pre metabolic syndrome
PRS: Polygenic risk score
BMI: Body mass index
SBP: Systolic blood pressure
DBP: Diastolic blood pressure
WC: Waist circumference
HbA1c: Glycated haemoglobin
HDL-C: High-density lipoprotein cholesterol
TG: Triglyceride
HR: Hazard ratio
CI: Confidence interval
ICD: International Classification of Diseases
SD: Standard deviation
UKB: UK Biobank
