# Supplementary material for: Association between metabolic syndrome and kidney cancer risk: a prospective cohort study
Source: Lipids Health Dis. 2024 May 17;23:142. doi: 10.1186/s12944-024-02138-5 (PMC11100063; doi:10.1186/s12944-024-02138-5)
Supplement: Supplementary file 1 — Supplementary Material 1. [file 12944_2024_2138_MOESM1_ESM.docx]

**Association between metabolic syndrome and kidney cancer risk:** **a prospective cohort study**

Lin Wang, Han Du, Chao Sheng, Hongji Dai* and Kexin Chen*

**Table of contents:**

Table S1. Detailed information of SNPs for PRS calculation.

Table S2. Baseline characteristics of the participants with complete or missing MetS components.

Table S3. Associations between MetS and kidney cancer risk by excluding participants followed <2 years.

Table S4. Associations between MetS and kidney cancer risk by excluding participants with outliers.

Table S5. Associations between MetS and kidney cancer risk by excluding participants with diabetes.

Table S6. Associations between MetS and kidney cancer risk by excluding cases identified through death registry records.

Table S7. Adjusted HRs for kidney cancer with MetS component combinations by excluding participants followed <2 years.

Table S8. Adjusted HRs for kidney cancer with MetS component combinations by excluding participants with outliers.

Figure S1. Flow diagram of the study design.

Figure S2. Proportion of metabolic syndrome (MetS) and its components in the study population.

Figure S3. Association of hypertriglyceridemia with risk of kidney cancer stratified by different subgroups.

Figure S4. Association of central obesity with risk of kidney cancer stratified by different subgroups.

Figure S5. Association of hyperglycemia with risk of kidney cancer stratified by different subgroups.

Figure S6. Association of dyslipidemia with risk of kidney cancer stratified by different subgroups.

Figure S7. Association of hypertension with risk of kidney cancer stratified by different subgroups.

**Table S1. Detailed information of SNPs for PRS calculation.**

| **Cancer** | **Source** | **rsID** | **Chr** | **Pos(hg19)** | **Risk Allele** | **PRS weight** |
| --- | --- | --- | --- | --- | --- | --- |
| Kidney | Scelo 2017 | rs4381241 | 1 | 50907438 | C | 0.10 |
| Kidney | Henrion 2015 | rs3845536 | 1 | 165650787 | C | 0.19 |
| Kidney | Scelo 2017 | rs11894252 | 2 | 46533376 | T | 0.15 |
| Kidney | Henrion 2015 | rs4953346 | 2 | 46558208 | G | 0.21 |
| Kidney | Scelo 2017 | rs6755594 | 2 | 46589295 | G | 0.10 |
| Kidney | Scelo 2017 | rs12105918 | 2 | 145208193 | C | 0.22 |
| Kidney | Scelo 2017 | rs67311347 | 3 | 40533243 | G | 0.10 |
| Kidney | Scelo 2017 | rs10936602 | 3 | 169536637 | T | 0.10 |
| Kidney | Scelo 2017 | rs234043 | 3 | 172313367 | C | 0.11 |
| Kidney | Scelo 2017 | rs7697932 | 4 | 101005318 | G | 0.11 |
| Kidney | Scelo 2017 | rs2241261 | 8 | 22876739 | T | 0.10 |
| Kidney | Scelo 2017 | rs11813268 | 10 | 105682296 | T | 0.11 |
| Kidney | Scelo 2017 | rs7105934 | 11 | 69239741 | G | 0.36 |
| Kidney | Scelo 2017 | rs74911261 | 11 | 108357137 | A | 0.34 |
| Kidney | Scelo 2017 | rs718314 | 12 | 26453283 | G | 0.17 |
| Kidney | Scelo 2017 | rs4765623 | 12 | 125320850 | T | 0.14 |
| Kidney | Scelo 2017 | rs8007348 | 14 | 73272730 | A | 0.11 |
| Kidney | Scelo 2017 | rs4903064 | 14 | 73279420 | C | 0.19 |
| Kidney | Scelo 2017 | rs714024 | 22 | 47013535 | C | 0.15 |

**Table S2. Baseline characteristics of the participants with complete or missing MetS components.**

| **Characteristic** | **Complete MetS components** | **Missing MetS components** |
| --- | --- | --- |
| N (%) | 374960 (80.9) | 88801 (19.1) |
| Age, mean (SD), years | 56.29 (8.1) | 56.18 (8.7) |
| Gender, N (%) |  |  |
| Female | 198274 (52.9) | 49702 (56.0) |
| Male | 176686 (47.1) | 39098 (44.0) |
| NA | 0 (0.0) | 1 (0.0) |
| Ethnicity, N (%) |  |  |
| Nonwhite | 19126 (5.1) | 6664 (7.5) |
| White | 354095 (94.4) | 81273 (91.5) |
| NA | 1739 (0.5) | 864 (1.0) |
| Townsend deprivation index, mean (SD) | -0.1 (35.1) | 0.2 (36.4) |
| Education level, N (%) |  |  |
| College or university degree | 121741 (32.5) | 28053 (31.6) |
| Others | 248817 (66.3) | 55818 (62.9) |
| NA | 4402 (1.2) | 4930 (5.5) |
| Smoking status, N (%) |  |  |
| Never | 205097 (54.7) | 49102 (55.3) |
| Former | 128361 (34.2) | 29551 (33.2) |
| Current | 39671 (10.6) | 9286 (10.5) |
| NA | 1831 (0.5) | 862 (1.0) |
| Drinking status, N (%) |  |  |
| Never | 16206 (4.3) | 4420 (5.0) |
| Former | 13104 (3.5) | 3245 (3.7) |
| Current | 344745 (91.9) | 80495 (90.6) |
| NA | 905 (0.3) | 641 (0.7) |
| BMI category, N (%) |  |  |
| <25 kg/m^2^ | 123339 (32.9) | 28814 (32.4) |
| 25-29.9 kg/m^2^ | 160015 (42.7) | 36202 (40.8) |
| ≥30 kg/m^2^ | 90857 (24.2) | 21646 (24.4) |
| NA | 749 (0.2) | 2139 (2.4) |
| WC, mean (SD), cm | 90.4 (13.4) | 90.3 (13.7) |
| SBP, mean (SD), mm Hg | 137.8 (18.6) | 137.5 (18.8) |
| DBP, mean (SD), mm Hg | 82.3 (10.1) | 82.0 (10.2) |
| HDL-C, mean (SD), mmol/l | 1.5 (0.4) | 1.44 (0.4) |
| Triglyceride, mean (SD), mmol/l | 1.8 (1.0) | 1.7 (1.0) |
| HbA1C, mean (SD), mmol/mol | 36.1 (6.8) | 36.2 (7.0) |
| Statins (Yes, %) | 60422 (16.1) | 14109 (15.9) |
| Diabetes medications (Yes, %) | 8435 (2.2) | 2232 (2.5) |
| Hypertension medications (Yes, %) | 76639 (20.4) | 17641 (19.9) |

Note: Participants with complete or missing MetS components further excluded missing covariates.

**Table S3. Associations between MetS and kidney cancer risk by excluding participants followed <2 years.**

| **MetS or component** | **Cases/Person-years** | **Incidence rates(per 100,000)** | **HR (95% CI)** | | | | | | |  |
| --- | --- | --- | --- | --- | --- | --- | --- | --- | --- | --- |
|  |  |  | **Crude** | ***P*** | **Model1** | ***P*** | **Model2** | ***P*** | **Model3** | ***P*** |
| **MetS** |  |  |  |  |  |  |  |  |  |  |
| No | 617/2,823,833 | 21.85 | Reference |  | Reference |  | Reference |  | Reference |  |
| Yes | 451/1,060,858 | 42.51 | 1.95 (1.73-2.21) | <0.001 | 1.61 (1.42-1.82) | <0.001 | 1.55 (1.37-1.76) | <0.001 | 1.25 (1.08-1.44) | 0.003 |
| **MetS stage** |  |  |  |  |  |  |  |  |  |  |
| Metabolically healthy | 70/632,386 | 11.07 | Reference |  | Reference |  | Reference |  | Reference |  |
| pre-MetS | 547/2,191,447 | 24.96 | 2.26 (1.76-2.90) | <0.001 | 1.47 (1.14-1.89) | 0.003 | 1.43 (1.11-1.84) | 0.006 | 1.28 (0.99-1.65) | 0.062 |
| MetS | 451/1,060,859 | 42.51 | 3.86 (3.00-4.97) | <0.001 | 2.26 (1.74-2.92) | <0.001 | 2.13 (1.64-2.76) | <0.001 | 1.57 (1.18-2.08) | 0.002 |
| **Hypertension** |  |  |  |  |  |  |  |  |  |  |
| No | 185/1,209,177 | 15.30 | Reference |  | Reference |  | Reference |  | Reference |  |
| Yes | 883/2,675,514 | 33.00 | 2.16 (1.85-2.53) | <0.001 | 1.39 (1.18-1.64) | <0.001 | 1.36 (1.15-1.60) | <0.001 | 1.19 (1.01-1.41) | 0.039 |
| **Central obesity** |  |  |  |  |  |  |  |  |  |  |
| No | 583/2,617,226 | 22.28 | Reference |  | Reference |  | Reference |  | Reference |  |
| Yes | 485/1,267,465 | 38.27 | 1.72 (1.53-1.94) | <0.001 | 1.66 (1.47-1.87) | <0.001 | 1.59 (1.40-1.79) | <0.001 | 1.20 (1.02-1.42) | 0.027 |
| **Dyslipidemia** |  |  |  |  |  |  |  |  |  |  |
| No | 750/3,105,787 | 24.15 | Reference |  | Reference |  | Reference |  | Reference |  |
| Yes | 318/778,905 | 40.83 | 1.69 (1.48-1.93) | <0.001 | 1.84 (1.61-2.09) | <0.001 | 1.81 (1.58-2.06) | <0.001 | 1.60 (1.39-1.83) | <0.001 |
| **Hypertriglyceridemia** |  |  |  |  |  |  |  |  |  |  |
| No | 409/2,033,197 | 20.12 | Reference |  | Reference |  | Reference |  | Reference |  |
| Yes | 659/1,851,495 | 35.59 | 1.77 (1.57-2.01) | <0.001 | 1.29 (1.14-1.47) | <0.001 | 1.25 (1.10-1.42) | <0.001 | 1.09 (0.96-1.25) | 0.189 |
| **Hyperglycemia** |  |  |  |  |  |  |  |  |  |  |
| No | 925/3,583,272 | 25.81 | Reference |  | Reference |  | Reference |  | Reference |  |
| Yes | 143/301,419 | 47.44 | 1.86 (1.56-2.21) | <0.001 | 1.40 (1.17-1.67) | <0.001 | 1.37 (1.14-1.64) | <0.001 | 1.13 (0.94-1.37) | 0.186 |
| **No. of MetS components** |  |  |  |  |  |  |  |  |  |  |
| 0 | 70/632,386 | 11.07 | Reference |  | Reference |  | Reference |  | Reference |  |
| 1 | 227/1,152,929 | 19.69 | 1.78 (1.36-2.33) | <0.001 | 1.26 (0.96-1.65) | 0.092 | 1.24 (0.95-1.63) | 0.115 | 1.18 (0.90-1.55) | 0.225 |
| 2 | 320/1,038,518 | 30.81 | 2.79 (2.16-3.62) | <0.001 | 1.68 (1.29-2.19) | <0.001 | 1.63 (1.25-2.12) | <0.001 | 1.44 (1.10-1.89) | 0.008 |
| 3 | 241/676,749 | 35.61 | 3.23 (2.48-4.22) | <0.001 | 1.91 (1.45-2.50) | <0.001 | 1.81 (1.38-2.38) | <0.001 | 1.48 (1.11-1.98) | 0.008 |
| 4 | 152/305,961 | 49.68 | 4.52 (3.40-5.99) | <0.001 | 2.69 (2.02-3.59) | <0.001 | 2.55 (1.91-3.41) | <0.001 | 1.94 (1.41-2.67) | <0.001 |
| 5 | 58/78,149 | 74.22 | 6.78 (4.79-9.61) | <0.001 | 3.83 (2.69-5.45) | <0.001 | 3.63 (2.54-5.18) | <0.001 | 2.60 (1.76-3.85) | <0.001 |
| *P* for trend |  |  | <0.001 |  | <0.001 |  | <0.001 |  | <0.001 |  |

Abbreviations: MetS, metabolic syndrome; HR, hazard ratio; 95%CI, 95% confidence interval; No, Number; Crude: Univariate cox regression; Model1: Adjusted for age and sex; Model2: Adjusted for age, sex, ethnicity, Townsend deprivation index, education level, smoking status, alcohol status and intakes of vegetables, fruit, fish, and red meat; Model3: Adjusted as model 2 plus body mass index.

**Table S4. Associations between MetS and kidney cancer risk by excluding participants with outliers.**

| **MetS or component** | **Cases/Person-years** | **Incidence rates(per 100,000)** | **HR (95% CI)** | | | | | | |  |
| --- | --- | --- | --- | --- | --- | --- | --- | --- | --- | --- |
|  |  |  | **Crude** | ***P*** | **Model1** | ***P*** | **Model2** | ***P*** | **Model3** | ***P*** |
| **MetS** |  |  |  |  |  |  |  |  |  |  |
| No | 640/2,590,951 | 24.70 | Reference |  | Reference |  | Reference |  | Reference |  |
| Yes | 424/912,351 | 46.47 | 1.88 (1.67-2.13) | <0.001 | 1.58 (1.40-1.79) | <0.001 | 1.52 (1.34-1.72) | <0.001 | 1.27 (1.10-1.46) | 0.001 |
| **MetS stage** |  |  |  |  |  |  |  |  |  |  |
| Metabolically healthy | 71/552,466 | 12.85 | Reference |  | Reference |  | Reference |  | Reference |  |
| pre-MetS | 569/2,038,485 | 27.91 | 2.17 (1.70-2.78) | <0.001 | 1.44 (1.12-1.85) | 0.004 | 1.40 (1.09-1.80) | 0.008 | 1.27 (0.99-1.64) | 0.062 |
| MetS | 424/912,351 | 46.47 | 3.63 (2.82-4.66) | <0.001 | 2.18 (1.69-2.82) | <0.001 | 2.05 (1.58-2.65) | <0.001 | 1.59 (1.20-2.11) | 0.001 |
| **Hypertension** |  |  |  |  |  |  |  |  |  |  |
| No | 179/1,082,472 | 16.54 | Reference |  | Reference |  | Reference |  | Reference |  |
| Yes | 885/2,420,830 | 36.56 | 2.21 (1.89-2.60) | <0.001 | 1.45 (1.23-1.71) | <0.001 | 1.42 (1.20-1.68) | <0.001 | 1.28 (1.08-1.52) | 0.004 |
| **Central obesity** |  |  |  |  |  |  |  |  |  |  |
| No | 600/2,377,504 | 25.24 | Reference |  | Reference |  | Reference |  | Reference |  |
| Yes | 464/1,125,799 | 41.22 | 1.64 (1.45-1.85) | <0.001 | 1.60 (1.42-1.81) | <0.001 | 1.52 (1.35-1.72) | <0.001 | 1.21 (1.02-1.43) | 0.026 |
| **Dyslipidemia** |  |  |  |  |  |  |  |  |  |  |
| No | 781/2,839,892 | 27.50 | Reference |  | Reference |  | Reference |  | Reference |  |
| Yes | 283/663,410 | 42.66 | 1.55 (1.35-1.78) | <0.001 | 1.74 (1.52-1.99) | <0.001 | 1.71 (1.49-1.96) | <0.001 | 1.54 (1.34-1.78) | <0.001 |
| **Hypertriglyceridemia** |  |  |  |  |  |  |  |  |  |  |
| No | 431/1,840,767 | 23.41 | Reference |  | Reference |  | Reference |  | Reference |  |
| Yes | 633/1,662,535 | 38.07 | 1.63 (1.44-1.84) | <0.001 | 1.21 (1.06-1.37) | 0.003 | 1.16 (1.02-1.32) | 0.020 | 1.03 (0.91-1.18) | 0.607 |
| **Hyperglycemia** |  |  |  |  |  |  |  |  |  |  |
| No | 952/3,273,440 | 29.08 | Reference |  | Reference |  | Reference |  | Reference |  |
| Yes | 112/229,862 | 48.72 | 1.68 (1.38-2.05) | <0.001 | 1.26 (1.04-1.54) | 0.020 | 1.23 (1.01-1.50) | 0.044 | 1.06 (0.87-1.30) | 0.567 |
| **No. of MetS components** |  |  |  |  |  |  |  |  |  |  |
| 0 | 71/552,466 | 12.85 | Reference |  | Reference |  | Reference |  | Reference |  |
| 1 | 236/1,065,438 | 22.15 | 1.72 (1.32-2.25) | <0.001 | 1.24 (0.95-1.62) | 0.113 | 1.23 (0.94-1.60) | 0.137 | 1.17 (0.90-1.53) | 0.245 |
| 2 | 333/973,047 | 34.22 | 2.67 (2.06-3.45) | <0.001 | 1.64 (1.27-2.13) | <0.001 | 1.58 (1.22-2.06) | <0.001 | 1.42 (1.09-1.86) | 0.011 |
| 3 | 253/609,547 | 41.51 | 3.24 (2.49-4.21) | <0.001 | 1.96 (1.50-2.56) | <0.001 | 1.85 (1.41-2.42) | <0.001 | 1.54 (1.15-2.06) | 0.003 |
| 4 | 139/251,755 | 55.21 | 4.31 (3.24-5.74) | <0.001 | 2.65 (1.98-3.55) | <0.001 | 2.48 (1.85-3.33) | <0.001 | 1.96 (1.42-2.71) | <0.001 |
| 5 | 32/51,048 | 62.69 | 4.90 (3.23-7.44) | <0.001 | 2.86 (1.88-4.37) | <0.001 | 2.68 (1.75-4.09) | <0.001 | 2.03 (1.29-3.20) | 0.002 |
| *P* for trend |  |  | <0.001 |  | <0.001 |  | <0.001 |  | <0.001 |  |

Abbreviations: MetS, metabolic syndrome; HR, hazard ratio; 95%CI, 95% confidence interval; No, Number; Crude: Univariate cox regression; Model1: Adjusted for age and sex; Model2: Adjusted for age, sex, ethnicity, Townsend deprivation index, education level, smoking status, alcohol status and intakes of vegetables, fruit, fish, and red meat; Model3: Adjusted as model 2 plus body mass index.

**Table S5. Associations between MetS and kidney cancer risk by excluding participants with diabetes**.

| **MetS or component** | **Cases/Person-years** | **Incidence rates(per 100,000)** | **HR (95% CI)** | | | | | | |  |
| --- | --- | --- | --- | --- | --- | --- | --- | --- | --- | --- |
|  |  |  | **Crude** | ***P*** | **Model1** | ***P*** | **Model2** | ***P*** | **Model3** | ***P*** |
| **MetS** |  |  |  |  |  |  |  |  |  |  |
| No | 688/2,812,588 | 24.46146 | Reference |  | Reference |  | Reference |  | Reference |  |
| Yes | 434/923,398 | 47.00034 | 1.92 (1.71-2.17) | <0.001 | 1.61 (1.43-1.82) | <0.001 | 1.54 (1.36-1.74) | <0.001 | 1.27 (1.10-1.46) | 0.001 |
| **MetS stage** |  |  |  |  |  |  |  |  |  |  |
| Metabolically healthy | 74/632,519 | 11.69926 | Reference |  | Reference |  | Reference |  | Reference |  |
| pre-MetS | 614/2,180,070 | 28.16424 | 2.41 (1.89-3.07) | <0.001 | 1.54 (1.20-1.96) | 0.001 | 1.49 (1.17-1.90) | 0.001 | 1.35 (1.05-1.73) | 0.018 |
| MetS | 434/923,398 | 47.00034 | 4.03 (3.15-5.15) | <0.001 | 2.35 (1.83-3.03) | <0.001 | 2.19 (1.70-2.82) | <0.001 | 1.69 (1.28-2.22) | <0.001 |
| **Hypertension** |  |  |  |  |  |  |  |  |  |  |
| No | 191/1,193,609 | 16.00189 | Reference |  | Reference |  | Reference |  | Reference |  |
| Yes | 931/2,542,377 | 36.61928 | 2.29 (1.96-2.68) | <0.001 | 1.46 (1.24-1.71) | <0.001 | 1.43 (1.21-1.68) | <0.001 | 1.28 (1.08-1.51) | 0.003 |
| **Central obesity** |  |  |  |  |  |  |  |  |  |  |
| No | 642/2,568,999 | 24.99027 | Reference |  | Reference |  | Reference |  | Reference |  |
| Yes | 480/1,166,986 | 41.13159 | 1.65 (1.46-1.85) | <0.001 | 1.60 (1.42-1.80) | <0.001 | 1.52 (1.35-1.71) | <0.001 | 1.17 (0.99-1.38) | 0.052 |
| **Dyslipidemia** |  |  |  |  |  |  |  |  |  |  |
| No | 810/3,026,590 | 26.76279 | Reference |  | Reference |  | Reference |  | Reference |  |
| Yes | 312/709,395 | 43.98113 | 1.64 (1.44-1.87) | <0.001 | 1.83 (1.60-2.08) | <0.001 | 1.79 (1.57-2.04) | <0.001 | 1.61 (1.41-1.85) | <0.001 |
| **Hypertriglyceridemia** |  |  |  |  |  |  |  |  |  |  |
| No | 464/2,015,600 | 23.02044 | Reference |  | Reference |  | Reference |  | Reference |  |
| Yes | 658/1,720,386 | 38.24723 | 1.66 (1.48-1.87) | <0.001 | 1.21 (1.07-1.36) | 0.002 | 1.16 (1.03-1.31) | 0.018 | 1.03 (0.91-1.17) | 0.638 |
| **Hyperglycemia** | /0 |  |  |  |  |  |  |  |  |  |
| No | 1045/3,584,630 | 29.15224 | Reference |  | Reference |  | Reference |  | Reference |  |
| Yes | 77/151,356 | 50.87348 | 1.75 (1.39-2.21) | <0.001 | 1.33 (1.05-1.68) | 0.016 | 1.27 (1.00-1.60) | 0.046 | 1.09 (0.86-1.39) | 0.461 |
| **No.MetS components** |  |  |  |  |  |  |  |  |  |  |
| 0 | 74/632,518 | 11.69926 | Reference |  | Reference |  | Reference |  | Reference |  |
| 1 | 256/1,151,219 | 22.2373 | 1.90 (1.47-2.46) |  | 1.32 (1.02-1.72) |  | 1.30 (1.00-1.69) |  | 1.25 (0.96-1.62) |  |
| 2 | 358/1,028,851 | 34.7961 | 2.98 (2.32-3.83) |  | 1.76 (1.36-2.27) |  | 1.68 (1.30-2.17) |  | 1.51 (1.16-1.96) |  |
| 3 | 273/642,470 | 42.49223 | 3.64 (2.82-4.71) |  | 2.12 (1.63-2.76) |  | 1.99 (1.53-2.59) |  | 1.64 (1.24-2.18) |  |
| 4 | 138/250,467 | 55.09702 | 4.72 (3.56-6.26) |  | 2.87 (2.15-3.82) |  | 2.66 (2.00-3.55) |  | 2.07 (1.51-2.85) |  |
| 5 | 23/30,460 | 75.50938 | 6.48 (4.06-10.4) |  | 3.68 (2.29-5.89) |  | 3.35 (2.09-5.37) |  | 2.50 (1.52-4.11) |  |
| *P* for trend |  |  | <0.001 |  | <0.001 |  | <0.001 |  | <0.001 |  |

Abbreviations: MetS, metabolic syndrome; HR, hazard ratio; 95%CI, 95% confidence interval; No, Number; Crude: Univariate cox regression; Model1: Adjusted for age and sex; Model2: Adjusted for age, sex, ethnicity, Townsend deprivation index, education level, smoking status, alcohol status and intakes of vegetables, fruit, fish, and red meat; Model3: Adjusted as model 2 plus body mass index.

**Table S6. Associations between MetS and kidney cancer risk by excluding cases identified through death registries records.**

| **MetS or component** | **Cases/Person-years** | **Incidence rates(per 100,000)** | **HR (95% CI)** | | | | | | |  |
| --- | --- | --- | --- | --- | --- | --- | --- | --- | --- | --- |
|  |  |  | **Crude** | ***P*** | **Model1** | ***P*** | **Model2** | ***P*** | **Model3** | ***P*** |
| **MetS** |  |  |  |  |  |  |  |  |  |  |
| No | 684/2,824,700 | 24.21 | Reference |  | Reference |  | Reference |  | Reference |  |
| Yes | 505/1,061,488 | 47.57 | 1.97 (1.75-2.21) | <0.001 | 1.62 (1.44-1.82) | <0.001 | 1.55 (1.38-1.75) | <0.001 | 1.27 (1.10-1.45) | 0.001 |
| **MetS stage** |  |  |  |  |  |  |  |  |  |  |
| Normal | 73/632,511 | 11.54 | Reference |  | Reference |  | Reference |  | Reference |  |
| pre-MetS | 611/2,192,190 | 27.87 | 2.42 (1.90-3.08) | <0.001 | 1.57 (1.22-2.00) | <0.001 | 1.52 (1.19-1.95) | <0.001 | 1.37 (1.07-1.76) | 0.013 |
| MetS | 505/1,061,488 | 47.57 | 4.13 (3.23-5.28) | <0.001 | 2.40 (1.87-3.09) | <0.001 | 2.25 (1.75-2.90) | <0.001 | 1.71 (1.30-2.24) | <0.001 |
| **Hypertension** |  |  |  |  |  |  |  |  |  |  |
| No | 195/1,209,472 | 16.12 | Reference |  | Reference |  | Reference |  | Reference |  |
| Yes | 994/2,676,716 | 37.14 | 2.31 (1.98-2.69) | <0.001 | 1.48 (1.26-1.73) | <0.001 | 1.45 (1.23-1.70) | <0.001 | 1.28 (1.09-1.51) | 0.003 |
| **Central obesity** |  |  |  |  |  |  |  |  |  |  |
| No | 648/2,618,130 | 24.75 | Reference |  | Reference |  | Reference |  | Reference |  |
| Yes | 541/1,268,057 | 42.66 | 1.73 (1.54-1.94) | <0.001 | 1.65 (1.47-1.85) | <0.001 | 1.57 (1.40-1.77) | <0.001 | 1.22 (1.04-1.42) | 0.014 |
| **Dyslipidemia** |  |  |  |  |  |  |  |  |  |  |
| No | 829/3,106,861 | 26.68 | Reference |  | Reference |  | Reference |  | Reference |  |
| Yes | 360/779,327 | 46.19 | 1.73 (1.53-1.96) | <0.001 | 1.88 (1.66-2.12) | <0.001 | 1.84 (1.63-2.09) | <0.001 | 1.64 (1.44-1.87) | <0.001 |
| **Hypertriglyceridemia** |  |  |  |  |  |  |  |  |  |  |
| No | 467/2,033,808 | 22.96 | Reference |  | Reference |  | Reference |  | Reference |  |
| Yes | 722/1,852,380 | 38.98 | 1.70 (1.51-1.91) | <0.001 | 1.23 (1.10-1.39) | 0.001 | 1.19 (1.05-1.34) | 0.006 | 1.04 (0.92-1.18) | 0.540 |
| **Hyperglycemia** |  |  |  |  |  |  |  |  |  |  |
| No | 1035/3,584,515 | 28.87 | Reference |  | Reference |  | Reference |  | Reference |  |
| Yes | 154/301,673 | 51.05 | 1.78 (1.50-2.11) | <0.001 | 1.33 (1.12-1.58) | 0.001 | 1.3 (1.09-1.54) | 0.003 | 1.08 (0.90-1.29) | 0.401 |
| **No.MetS components** |  |  |  |  |  |  |  |  |  |  |
| 0 | 73/632,511 | 11.54 | Reference |  | Reference |  | Reference |  | Reference |  |
| 1 | 254/1,153,265 | 22.02 | 1.91 (1.47-2.48) |  | 1.35 (1.04-1.76) |  | 1.33 (1.02-1.73) |  | 1.27 (0.98-1.65) |  |
| 2 | 357/1,038,924 | 34.36 | 2.98 (2.32-3.84) |  | 1.79 (1.39-2.32) |  | 1.73 (1.33-2.23) |  | 1.54 (1.19-2.01) |  |
| 3 | 280/677,139 | 41.35 | 3.59 (2.78-4.65) |  | 2.11 (1.62-2.75) |  | 2.00 (1.53-2.60) |  | 1.65 (1.25-2.19) |  |
| 4 | 162/306,120 | 52.92 | 4.60 (3.49-6.07) |  | 2.73 (2.06-3.61) |  | 2.56 (1.93-3.40) |  | 1.99 (1.46-2.71) |  |
| 5 | 63/78,229 | 80.53 | 7.02 (5.01-9.84) |  | 3.94 (2.80-5.54) |  | 3.68 (2.61-5.20) |  | 2.71 (1.86-3.95) |  |
| P for trend |  |  | <0.001 |  | <0.001 |  | <0.001 |  | <0.001 |  |

Abbreviations: MetS, metabolic syndrome; HR, hazard ratio; 95%CI, 95% confidence interval; No, Number; Crude: Univariate cox regression; Model1: Adjusted for age and sex; Model2: Adjusted for age, sex, ethnicity, Townsend deprivation index, education level, smoking status, alcohol status and intakes of vegetables, fruit, fish, and red meat; Model3: Adjusted as model 2 plus body mass index.

**Table S7. Adjusted HRs for kidney cancer with MetS component combinations by excluding participants followed <2 years.**

| **No. of MetS** | **Cases/Person-years** | **Incidence rates (per 100,000)** | **Combinations of MetS components** | **Adjusted HR (95% CI)** | ***P*** |
| --- | --- | --- | --- | --- | --- |
| Metabolically healthy | 70/632,386 | 11.07 | None | Reference |  |
| (0 component) |  |  |  |  |  |
| pre-MetS(1-2) | 167/805,903 | 20.72 | BP | 1.16 (0.87-1.54) | 0.313 |
| (1-2 components) | 12/71,250 | 16.84 | HDL | 1.95 (1.06-3.61) | 0.033 |
|  | 28/183,025 | 15.30 | TG | 0.90 (0.58-1.39) | 0.627 |
|  | 19/85,618 | 22.19 | WC | 1.61 (0.96-2.71) | 0.071 |
|  | 17/56,552 | 30.06 | BP + HDL | 2.01 (1.18-3.42) | 0.010 |
|  | 184/558,717 | 32.93 | BP + TG | 1.35 (1.02-1.80) | 0.039 |
|  | 79/244,504 | 32.31 | BP + WC | 1.52 (1.07-2.15) | 0.019 |
|  | 14/59,145 | 23.67 | HDL + TG | 1.63 (0.92-2.90) | 0.095 |
|  | 17/62,851 | 27.05 | TG + WC | 1.43 (0.83-2.47) | 0.194 |
|  |  |  |  |  |  |
| MetS(3-5) | 63/141,117 | 44.64 | BP + HDL + TG | 2.00 (1.41-2.83) | <0.001 |
| (3-5 components) | 21/54,006 | 38.88 | BP + HDL + WC | 2.37 (1.42-3.96) | 0.001 |
|  | 123/355,126 | 34.64 | BP + WC + TG | 1.28 (0.92-1.77) | 0.145 |
|  | 12/48,600 | 24.69 | HDL + WC + TG | 1.57 (0.84-2.94) | 0.159 |
|  | 11/47,248 | 23.28 | BP + TG + HbA1c | 0.81 (0.43-1.54) | 0.524 |
|  | 96/196,555 | 48.84 | BP + HDL + WC +TG | 2.09 (1.48-2.95) | <0.001 |
|  | 36/73,527 | 48.96 | BP + WC + TG + HbA1c | 1.50 (0.97-2.34) | 0.071 |
|  | 58/78,149 | 74.22 | BP + HDL + WC + TG + HbA1c | 2.59 (1.73-3.86) | <0.001 |

Note: only combinations with >10 events were presented.

Abbreviations: pre-MetS: metabolic syndrome; MetS, metabolic syndrome; BP, blood pressure; HDL, high-density lipoprotein cholesterol; TG, triglycerides; WC, waist circumference; No., Number; HR, hazard ratio; 95%CI, 95% confidence interval.

HR was adjusted for age, sex, ethnicity, Townsend deprivation index, education level, smoking status, alcohol status, intakes of vegetables, fruit, fish, and red meat and body mass index.

**Table S8. Adjusted HRs for kidney cancer with MetS component combinations by excluding participants with outliers.**

| **No. of MetS** | **Cases/Person-years** | **Incidence rates (per 100,000)** | **Combinations of MetS components** | **Adjusted HR (95% CI)** | ***P*** |
| --- | --- | --- | --- | --- | --- |
| Metabolically healthy | 71/552,466 | 12.85 | None | Reference |  |
| (0 component) |  |  |  |  |  |
| pre-MetS(1-2) | 179/742,082 | 24.12 | BP | 1.19 (0.90-1.58) | 0.216 |
| (1-2 components) | 10/62,891 | 15.90 | HDL | 1.61 (0.83-3.12) | 0.160 |
|  | 29/173,047 | 16.76 | TG | 0.87 (0.56-1.34) | 0.532 |
|  | 17/81,346 | 20.90 | WC | 1.34 (0.78-2.31) | 0.284 |
|  | 20/52,583 | 38.04 | BP + HDL | 2.28 (1.38-3.75) | 0.001 |
|  | 188/530,206 | 35.46 | BP + TG | 1.30 (0.98-1.73) | 0.070 |
|  | 89/227,443 | 39.13 | BP + WC | 1.66 (1.18-2.34) | 0.004 |
|  | 12/52,159 | 23.01 | HDL + TG | 1.42 (0.77-2.62) | 0.262 |
|  | 17/60,306 | 28.19 | TG + WC | 1.34 (0.78-2.31) | 0.292 |
|  |  |  |  |  |  |
| MetS(3-5) | 63/123,239 | 51.12 | BP + HDL + TG | 2.07 (1.46-2.93) | <0.001 |
| (3-5 components) | 24/49,785 | 48.21 | BP + HDL + WC | 2.71 (1.65-4.43) | <0.001 |
|  | 130/328,362 | 39.59 | BP + WC + TG | 1.32 (0.95-1.83) | 0.095 |
|  | 13/43,225 | 30.07 | HDL + WC + TG | 1.76 (0.96-3.23) | 0.068 |
|  | 13/39,482 | 32.93 | BP + TG + HbA1c | 0.99 (0.54-1.81) | 0.977 |
|  | 88/168,147 | 52.34 | BP + HDL + WC +TG | 2.07 (1.45-2.94) | <0.001 |
|  | 34/57,236 | 59.40 | BP + WC + TG + HbA1c | 1.64 (1.05-2.57) | 0.029 |
|  | 32/51,048 | 62.69 | BP + HDL + WC + TG + HbA1c | 2.05 (1.30-3.25) | 0.002 |

Note: only combinations with >10 events were presented.

Abbreviations: pre-MetS: metabolic syndrome; MetS, metabolic syndrome; BP, blood pressure; HDL, high-density lipoprotein cholesterol; TG, triglycerides; WC, waist circumference; No., Number; HR, hazard ratio; 95%CI, 95% confidence interval.

HR was adjusted for age, sex, ethnicity, Townsend deprivation index, education level, smoking status, alcohol status, intakes of vegetables, fruit, fish, and red meat and body mass index.

**
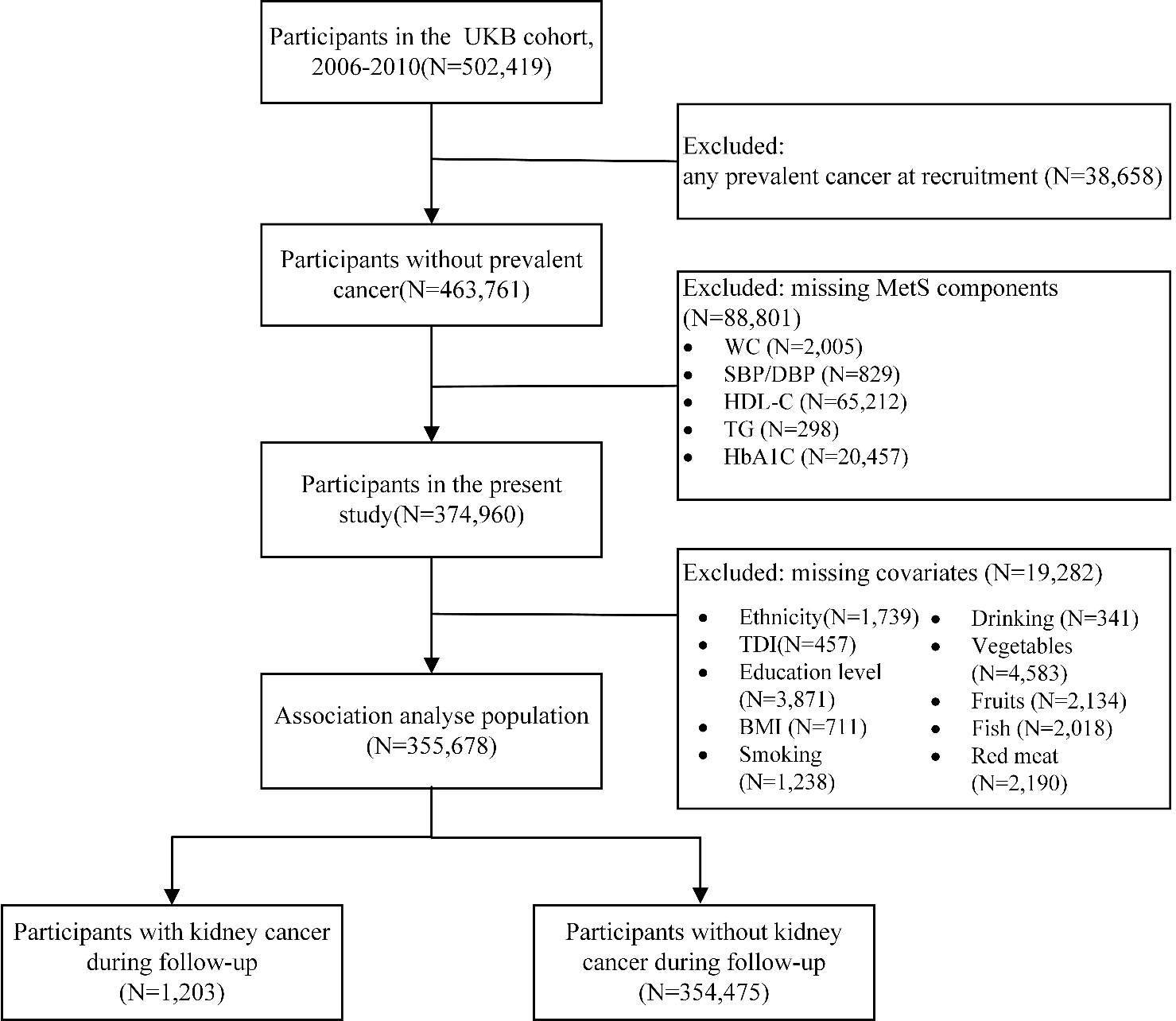
**

**Figure S1. Flow diagram of the study design.**

Abbreviations: MetS, metabolic syndrome; TDI, Townsend deprivation index; WC, waist circumference; SBP, systolic blood pressure; DBP, diastolic blood pressure; HDL-C, high-density lipoprotein cholesterol; TG, triglycerides; BMI, body mass index; N, Number

**
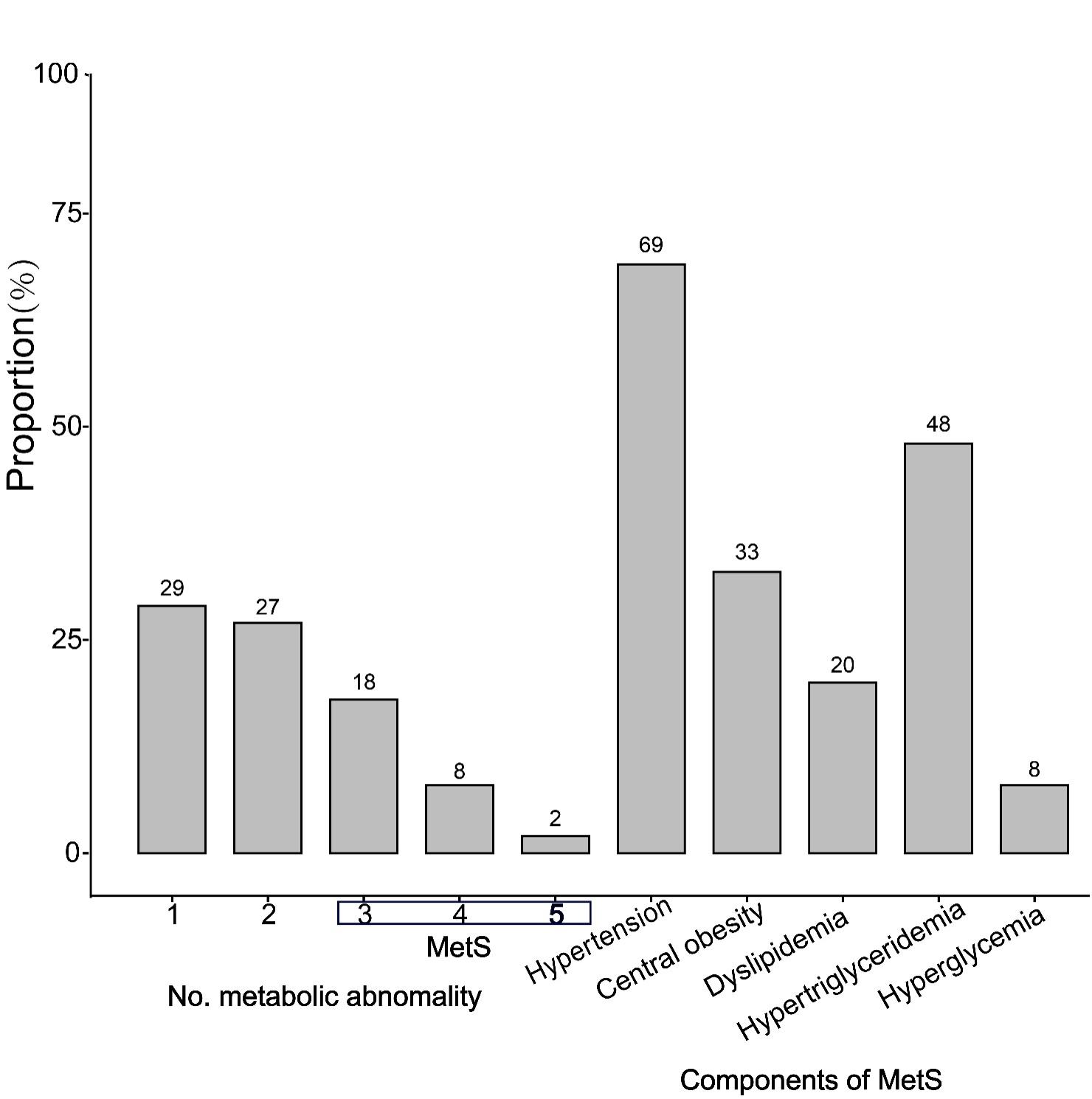
**

**Figure S2. Proportion of metabolic syndrome (MetS) and its components in the study population.**

Abbreviations: MetS, metabolic syndrome


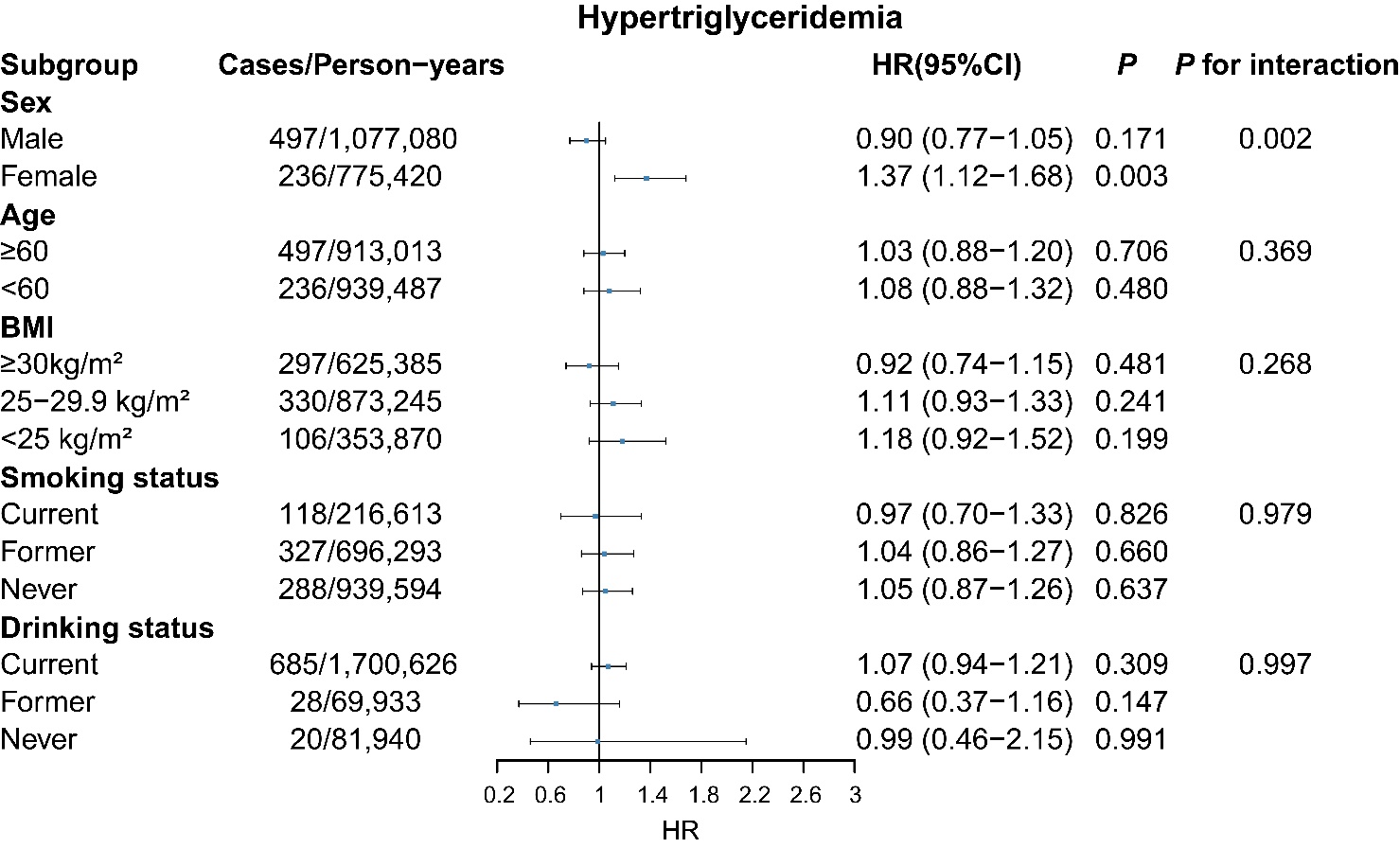


**Figure S3. Association of hypertriglyceridemia with risk of kidney cancer stratified by different subgroups.**

Note: Cases/Person-years in each subgroup correspond to the participants with MetS.

Abbreviations: MetS, metabolic syndrome; HR, hazard ratio; 95%CI,95% confidence interval.

Fully adjusted model incorporated age, sex, ethnicity, Townsend deprivation index, education level, smoking status, alcohol status, intakes of vegetables, fruit, fish, and red meat and body mass index. All subgroup analyses were performed using the fully adjusted model except stratified factor.


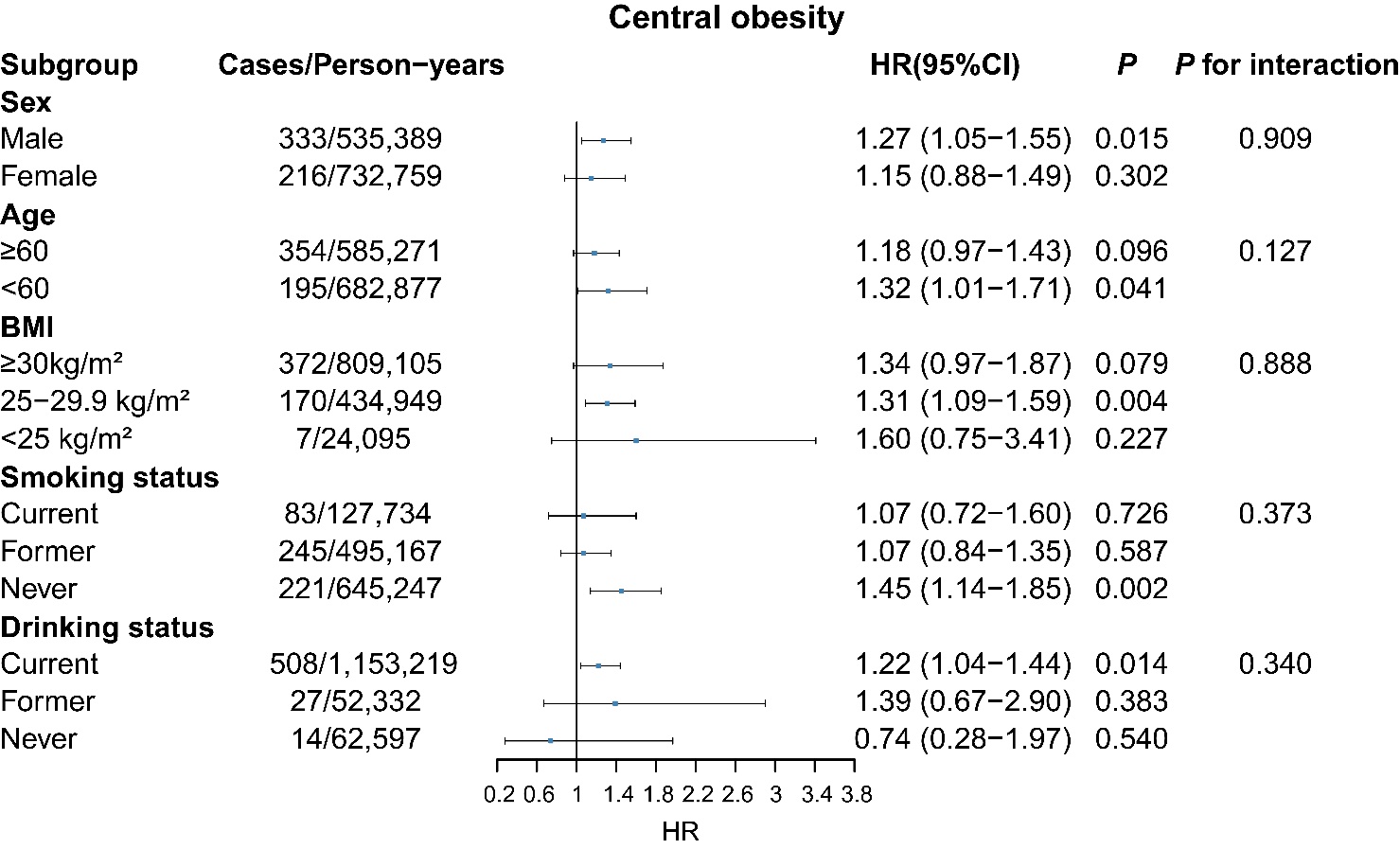


**Figure S4. Association of central obesity with risk of kidney cancer stratified by different subgroups.**

Note: Cases/Person-years in each subgroup correspond to the participants with MetS.

Abbreviations: MetS, metabolic syndrome; HR, hazard ratio; 95%CI,95% confidence interval.

Fully adjusted model incorporated age, sex, ethnicity, Townsend deprivation index, education level, smoking status, alcohol status, intakes of vegetables, fruit, fish, and red meat and body mass index. All subgroup analyses were performed using the fully adjusted model except stratified factor.


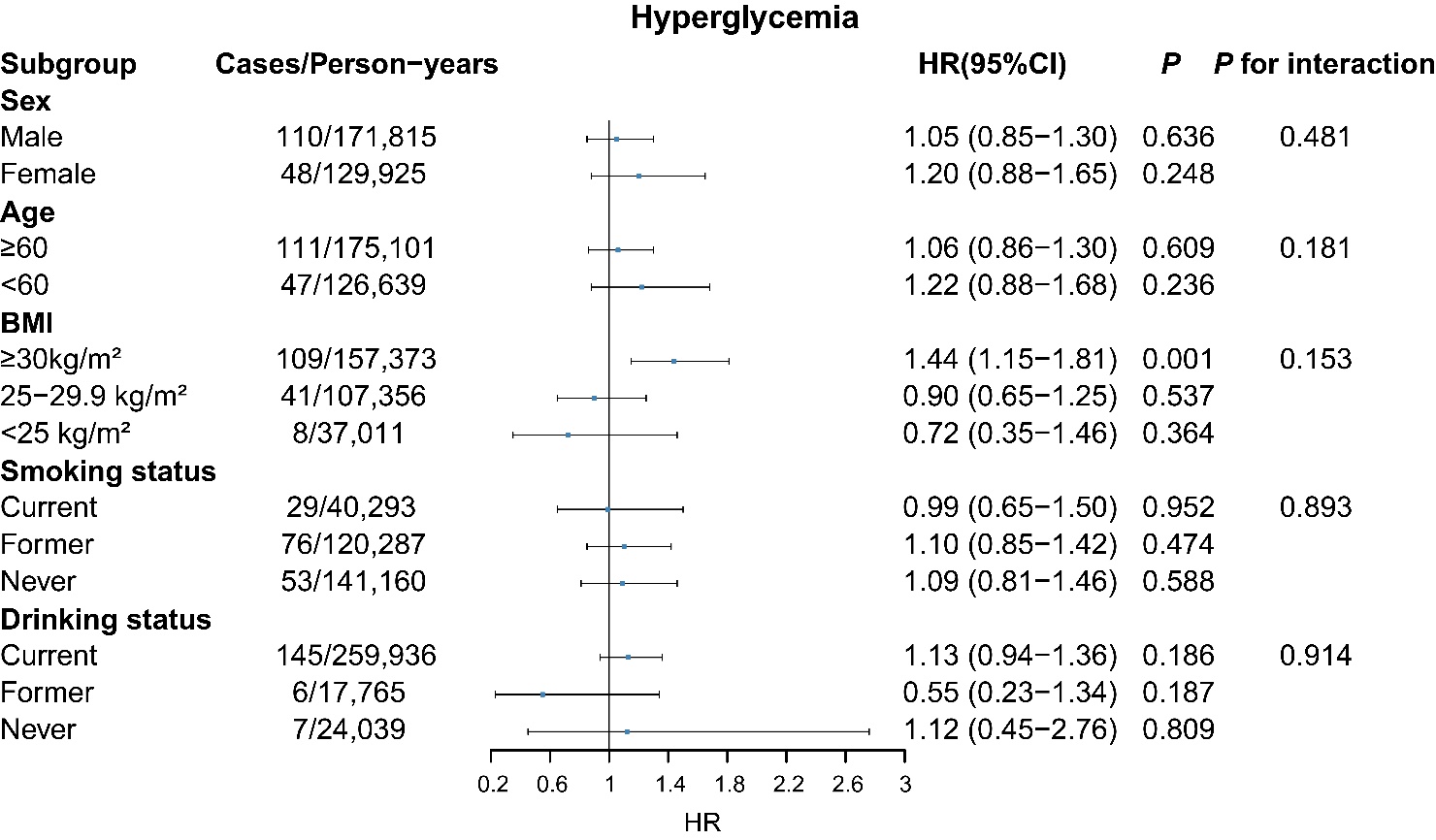


**Figure S5. Association of hyperglycemia with risk of kidney cancer stratified by different subgroups.**

Note: Cases/Person-years in each subgroup correspond to the participants with MetS.

Abbreviations: MetS, metabolic syndrome; HR, hazard ratio; 95%CI,95% confidence interval.

Fully adjusted model incorporated age, sex, ethnicity, Townsend deprivation index, education level, smoking status, alcohol status, intakes of vegetables, fruit, fish, and red meat and body mass index. All subgroup analyses were performed using the fully adjusted model except stratified factor.


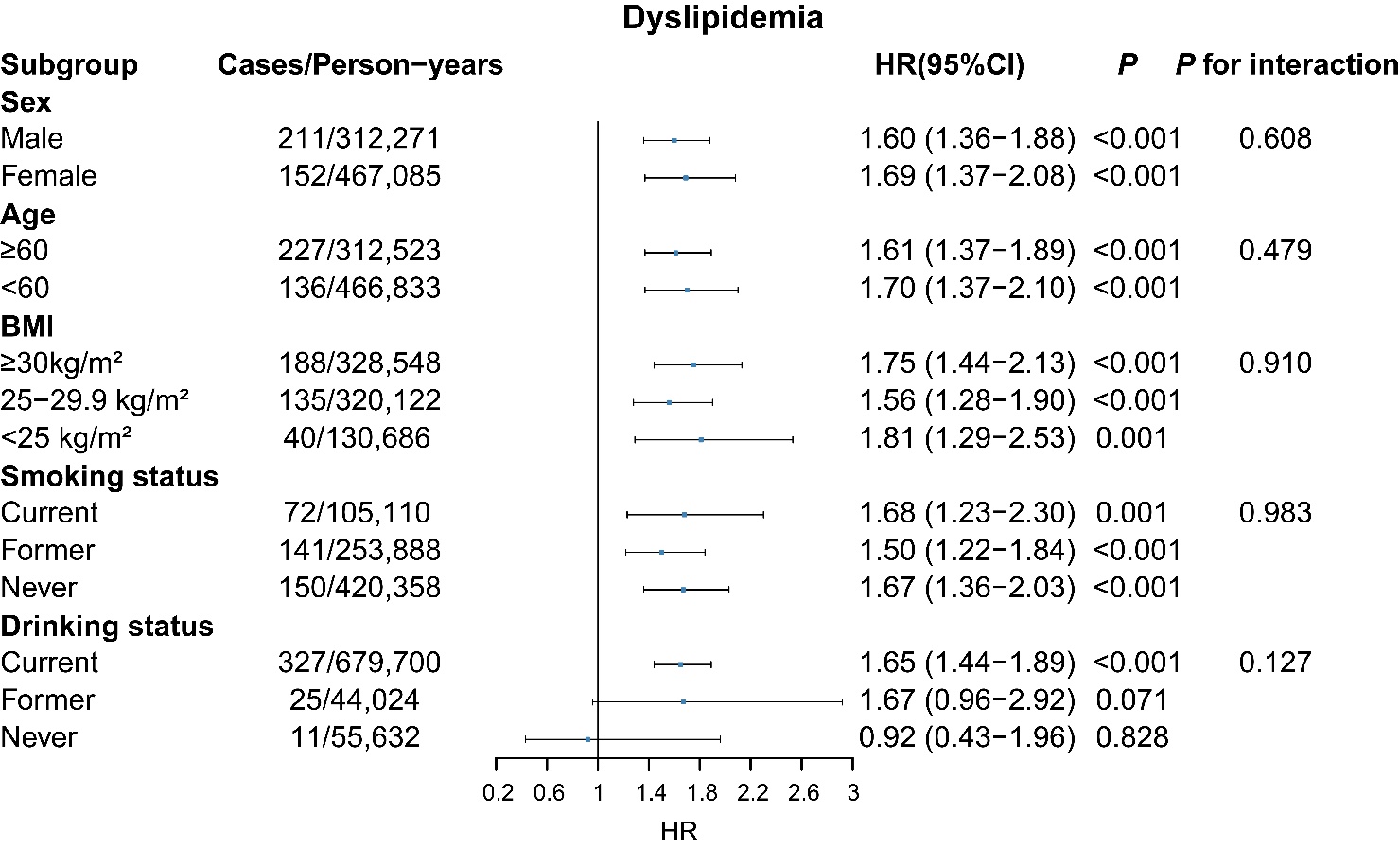


**Figure S6. Association of dyslipidemia with risk of kidney cancer stratified by different subgroups.**

Note: Cases/Person-years in each subgroup correspond to the participants with MetS.

Abbreviations: MetS, metabolic syndrome; HR, hazard ratio; 95%CI,95% confidence interval.

Fully adjusted model incorporated age, sex, ethnicity, Townsend deprivation index, education level, smoking status, alcohol status, intakes of vegetables, fruit, fish, and red meat and body mass index. All subgroup analyses were performed using the fully adjusted model except stratified factor.


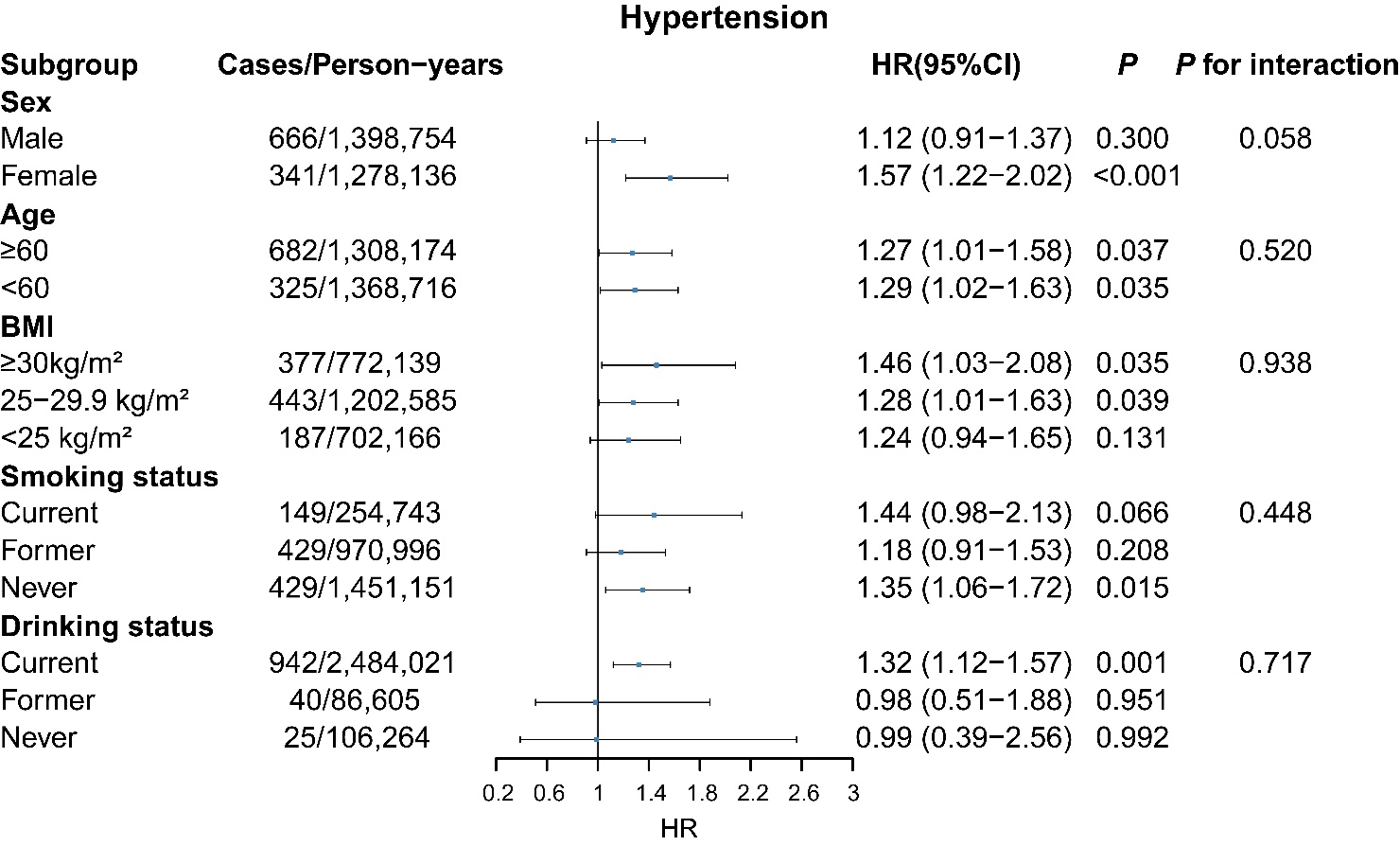


**Figure S7. Association of hypertension with risk of kidney cancer stratified by different subgroups.**

Note: Cases/Person-years in each subgroup correspond to the participants with MetS.

Abbreviations: MetS, metabolic syndrome; HR, hazard ratio; 95%CI,95% confidence interval.

Fully adjusted model incorporated age, sex, ethnicity, Townsend deprivation index, education level, smoking status, alcohol status, intakes of vegetables, fruit, fish, and red meat and body mass index. All subgroup analyses were performed using the fully adjusted model except stratified factor.
